# Supplementary material for: Nano-topology optimization for materials design with atom-by-atom control
Source: Nat Commun. 2020 Jul 27;11:3745. doi: 10.1038/s41467-020-17570-1 (PMC7385150; doi:10.1038/s41467-020-17570-1)
Supplement: Supplementary file 1 — Supplementary Information [file 41467_2020_17570_MOESM1_ESM.pdf]

Supplementary Information

Nano-topology optimization for materials design

with atom-by-atom control

Chen *et al*.

Supplementary Figures

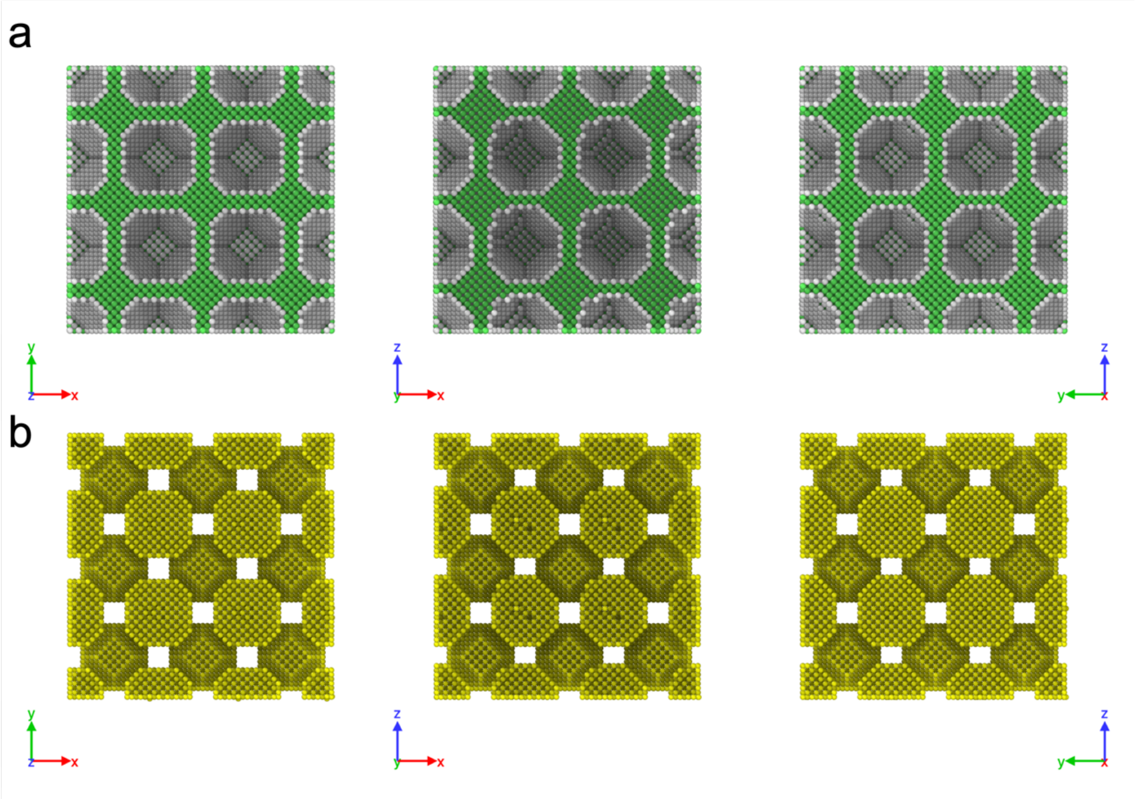

Supplementary Figure 1 | Nano-TO design for maximizing bulk modulus from different perspectives. a, Periodic images ($3 \times 3 \times 3$) of the Nano-TO design with a volume fraction of 50%. The bulk atoms are shown in green and the surface atoms are shown in grey. **b**, The corresponding distribution of virtual atoms. The surfaces in the Nano-TO design are mostly {1 1 1} and {1 0 0}. The virtual atoms form truncated octahedron structures in the BCC arrangement.

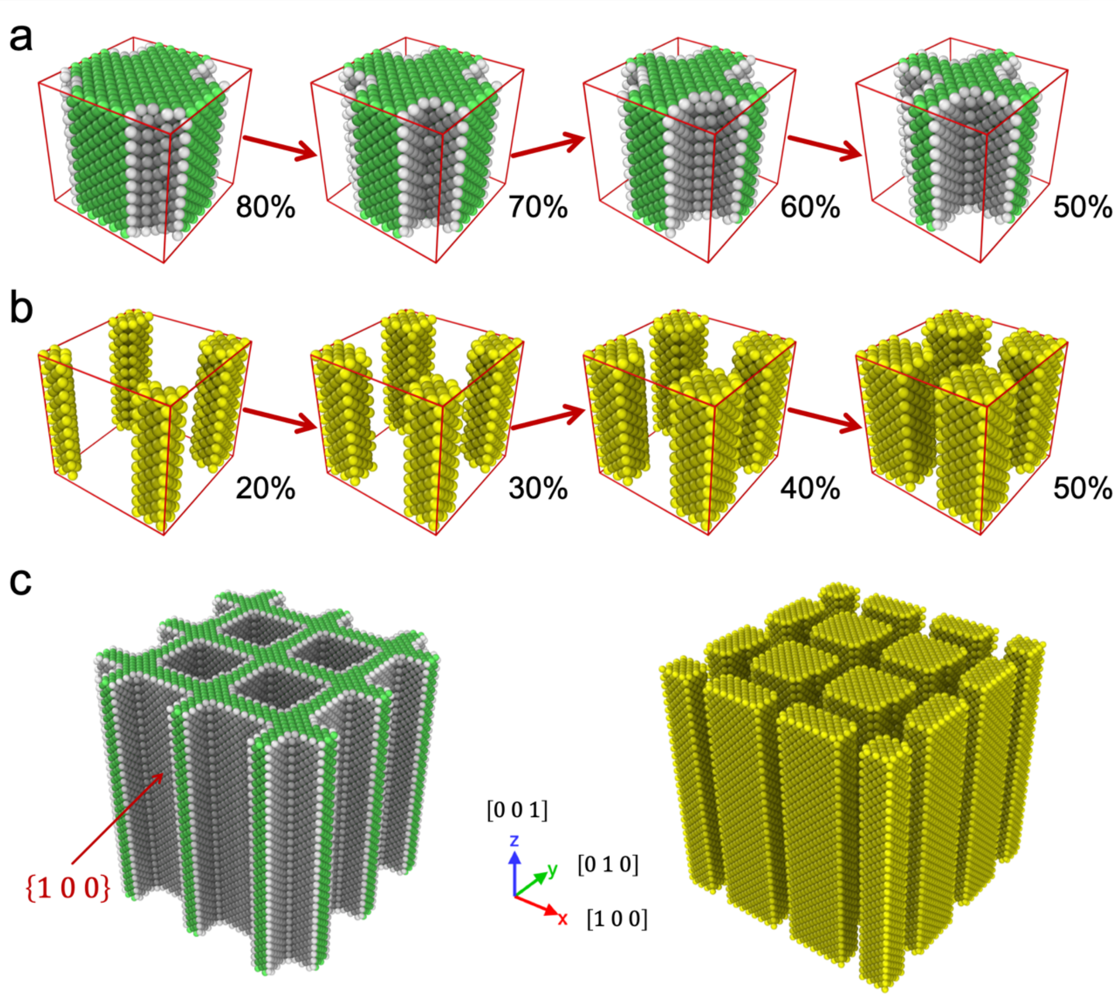

Supplementary Figure 2 | Nano-TO designs for maximizing elastic constant of C_{33}. a, The Nano-TO designs for maximizing the elastic constant of C_{33} with varying volume fractions from 50 to 80%. The bulk atoms are shown in green and the surface atoms are shown in grey. **b**, The corresponding distributions of the virtual atoms. **c**, Periodic images (3 × 3 × 3) of the Nano-TO design with a volume fraction of 50% and the corresponding distribution of virtual atoms. The surfaces in the Nano-TO designs are mostly {1 0 0}. The virtual atoms form pillar-like structures in parallel to the *z*-direction (i.e., [0 0 1]).

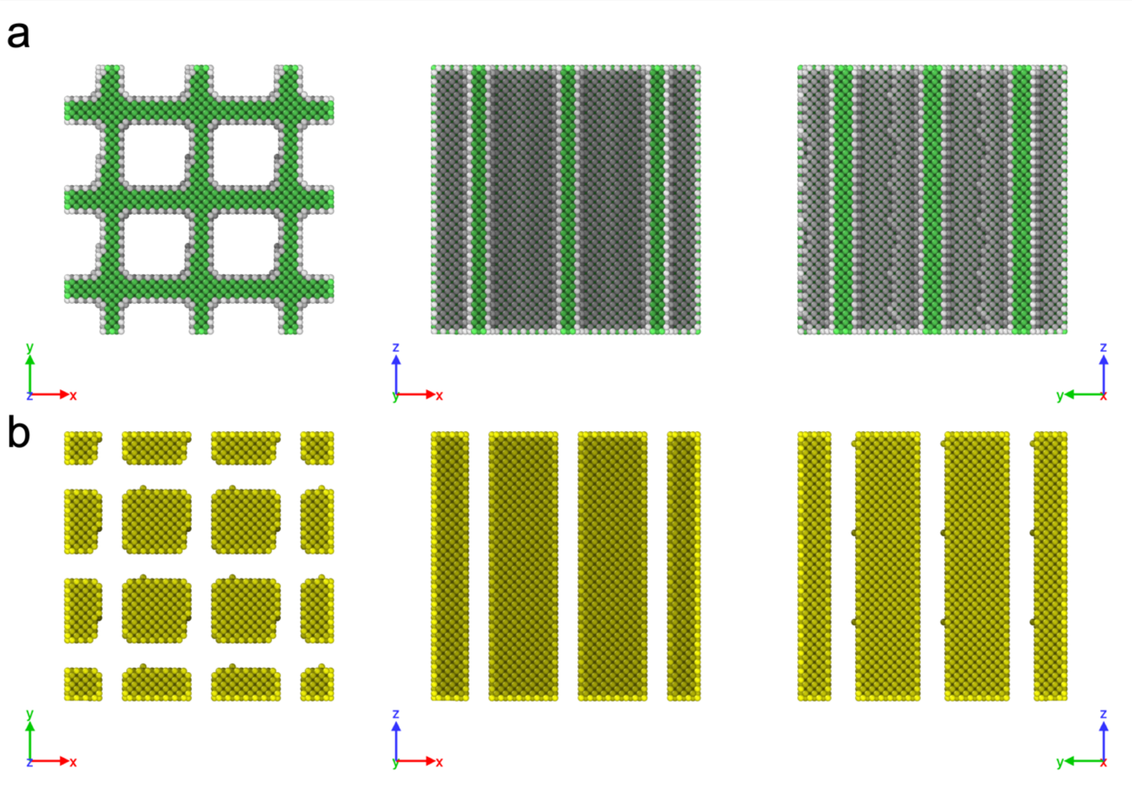

Supplementary Figure 3 | Nano-TO design for maximizing elastic constant of C_{33} from different perspectives. **a**, Periodic images ($3 \times 3 \times 3$) of the Nano-TO design with a volume fraction of 50%. The bulk atoms are shown in green and the surface atoms are shown in grey. **b**, The corresponding distribution of virtual atoms. The surfaces in the Nano-TO designs are mostly {1 0 0}. The virtual atoms form pillar-like structures in parallel to the *z*-direction (i.e., [0 0 1]).

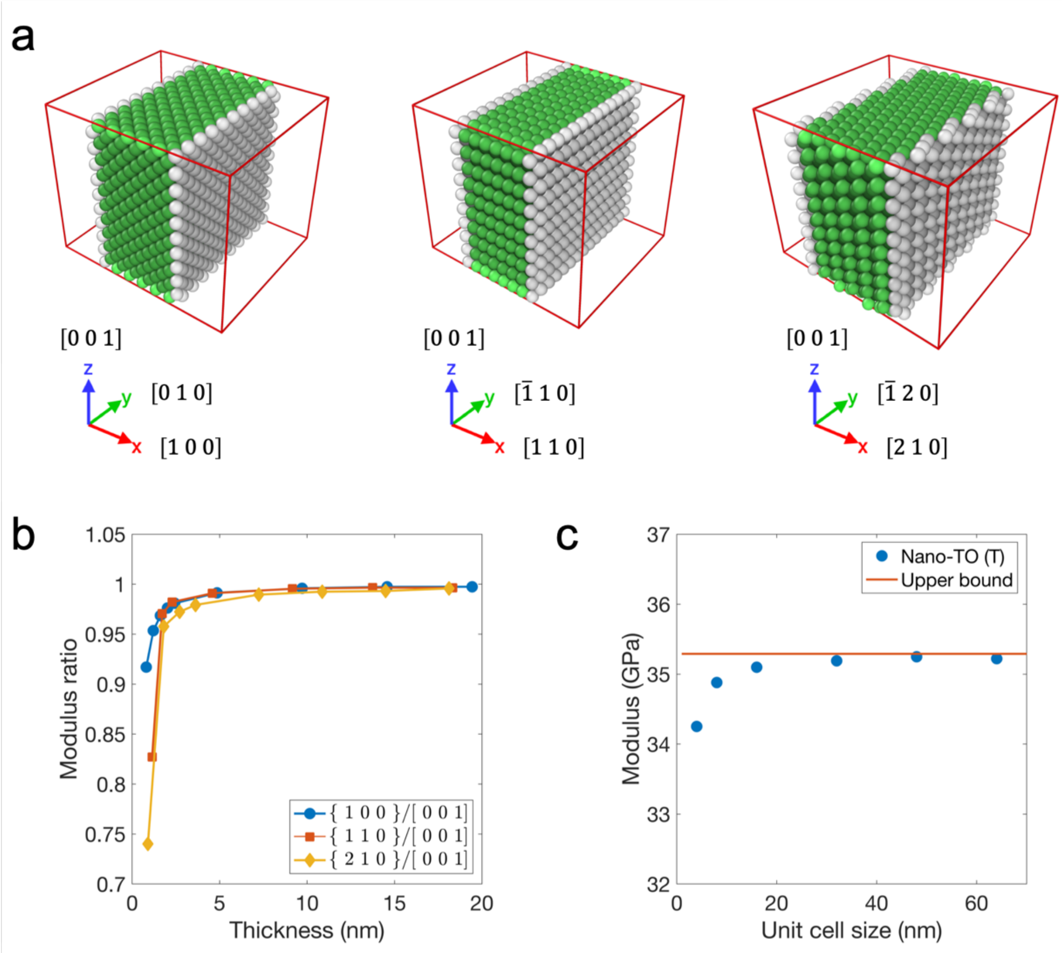

Supplementary Figure 4 | Surface effect in nanostructured material. **a**, The atomistic models of a nanoplate with surfaces {1 0 0}, {1 1 0}, and {2 1 0}, respectively. The bulk atoms are shown in green and the surface atoms are shown in grey. **b**, The modulus ratios in the *z*-direction (i.e., [0 0 1]) for nanoplates with varying thicknesses. The blue circles with solid line, red squares with solid line, and yellow diamonds with solid line represent the modulus ratios for nanoplates with surfaces {1 0 0}, {1 1 0}, and {2 1 0}, respectively. The modulus ratio is defined as the Young's modulus of a nanoplate divided by that of the bulk material. **c**, The Young's moduli of the Nano-TO designs (blue circles) with varying cell sizes in comparison with the theoretical upper bound. The volume fraction in the comparison is 50%.

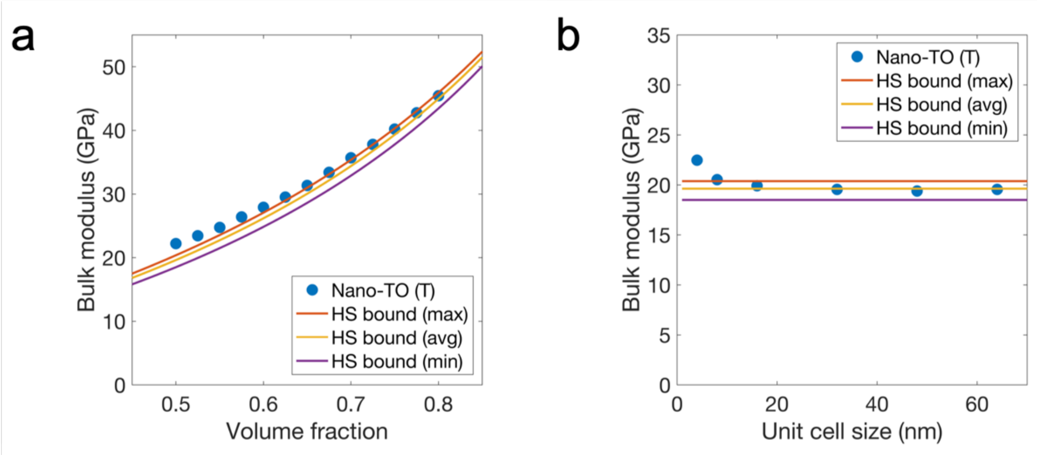

Supplementary Figure 5 | Comparison of Nano-TO designs and HS upper bounds. a, The bulk moduli of the Nano-TO designs (blue circles) with varying volume fractions in comparison with the HS upper bounds (red, yellow, and purple lines). To consider the anisotropy effect, the HS upper bounds are calculated with the maximum (31.60 GPa), average (29.28 GPa), and minimum (26.12 GPa) shear modulus, respectively. The length of the unit cells in the comparison is 4 nm. **b**, The bulk modulus of the Nano-TO design (blue circles) with varying cell sizes from 4 to 64 nm in comparison with the HS upper bounds (red, yellow, and purple lines). The volume fraction in the comparison is 50%.

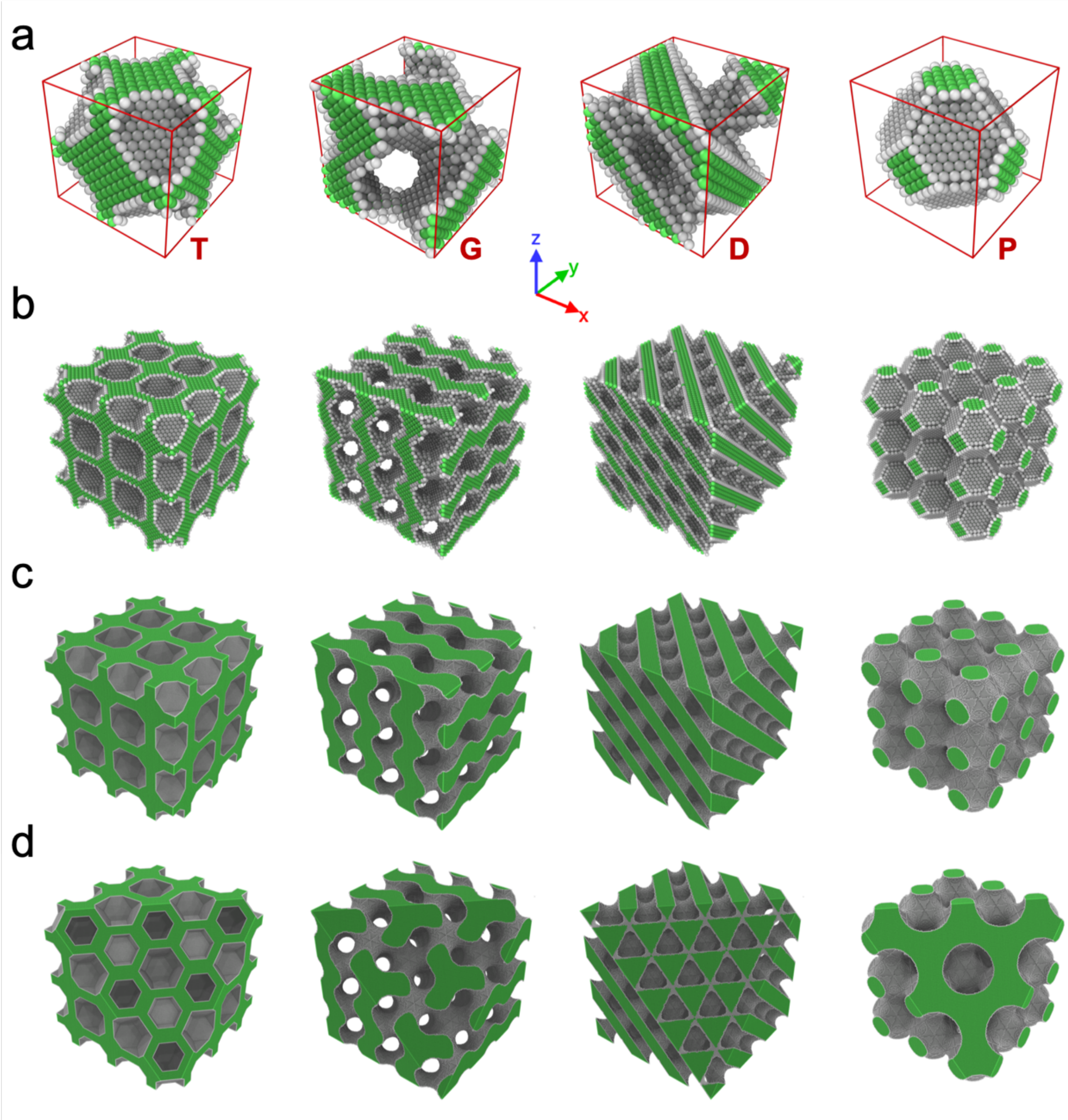

Supplementary Figure 6 | Nano-TO design and TPMS structures based on solid-networks. a, The unit cells of the Nano-TO design (T) for maximizing the bulk modulus and three TPMS structures including the gyroid (G), diamond (D), and primitive (P) with a volume fraction of 50%. The unit cells consist of approximately 2,000 atoms with a length of 4 nm. The bulk atoms are shown in green and the surface atoms are shown in grey. **b**, Periodic images (3 × 3 × 3) of the unit cells. **c**, Periodic images (3 × 3 × 3) of larger unit cells. The larger unit cells consist of approximately 128,000 atoms with a length of 16 nm. **d**, Slice views to reveal the interconnectivity.

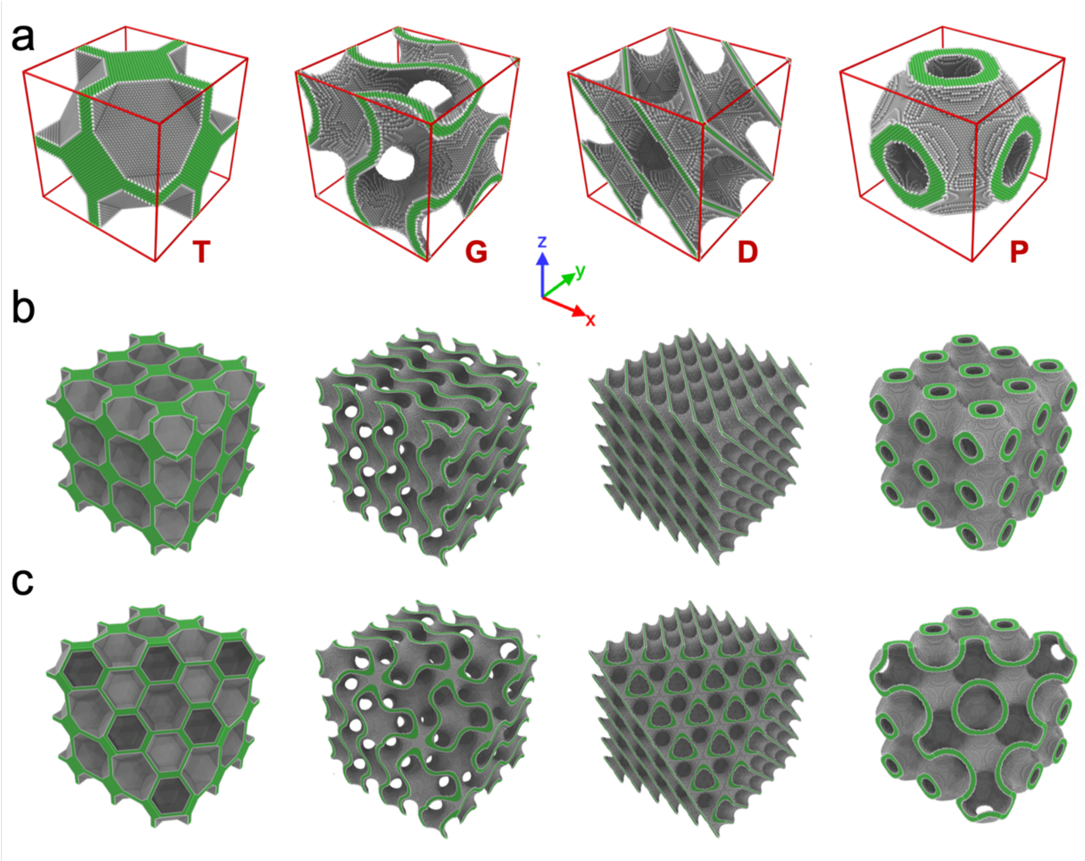

Supplementary Figure 7 | Nano-TO design and TPMS structures based on sheet-networks. a, The unit cells of the Nano-TO design (T) for maximizing the bulk modulus and three TPMS structures including the gyroid (G), diamond (D), and primitive (P) with a volume fraction of 25%. The unit cells consist of approximately 64,000 atoms with a length of 16 nm. The bulk atoms are shown in green and the surface atoms are shown in grey. **b**, Periodic images (3 × 3 × 3) of the unit cells. **c**, Slice views to reveal the interconnectivity.

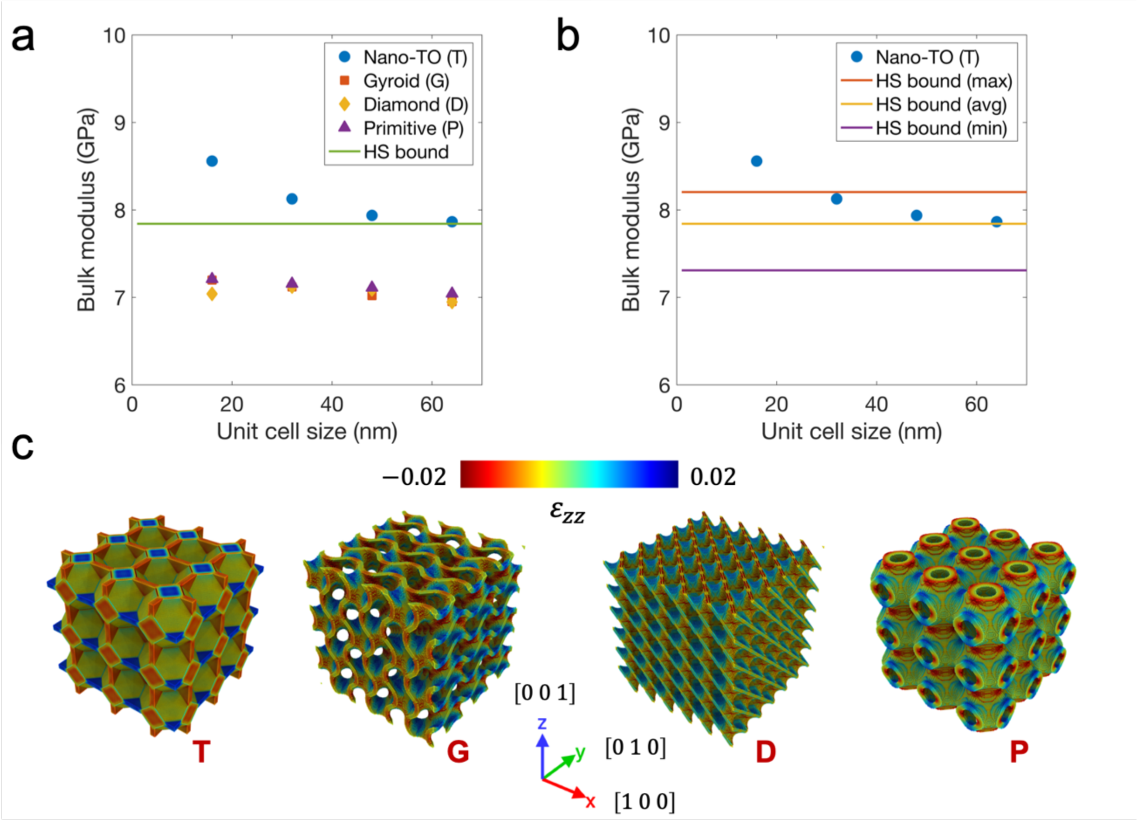

Supplementary Figure 8 | Performance of Nano-TO design and TPMS structures based on sheet-networks. a, The bulk moduli of the Nano-TO design (blue circles), gyroid structure (red squares), diamond structure (yellow diamonds), and primitive structure (purple triangles) with varying cell sizes from 16 to 64 nm in comparison with the HS upper bound (green line). The volume fraction in the comparison is 25%. **b,** The bulk modulus of the Nano-TO design (blue circles) in comparison with the HS upper bounds (red, yellow, and purple lines). To consider the anisotropy effect, the HS upper bounds are calculated with the maximum (31.60 GPa), average (29.28 GPa), and minimum (26.12 GPa) shear modulus, respectively. **c**, Atomic strain (ε_{zz}) distributions of those structures subjected to a constant hydrostatic (volumetric) strain of -10^{-2}. The cell size of those structures is 16 nm and the volume fraction is 25%. The average atomic strains for the Nano-TO design (T), gyroid (G), diamond (D), and primitive (P) are -0.0039, -0.0031, -0.0029, and -0.0031, respectively.

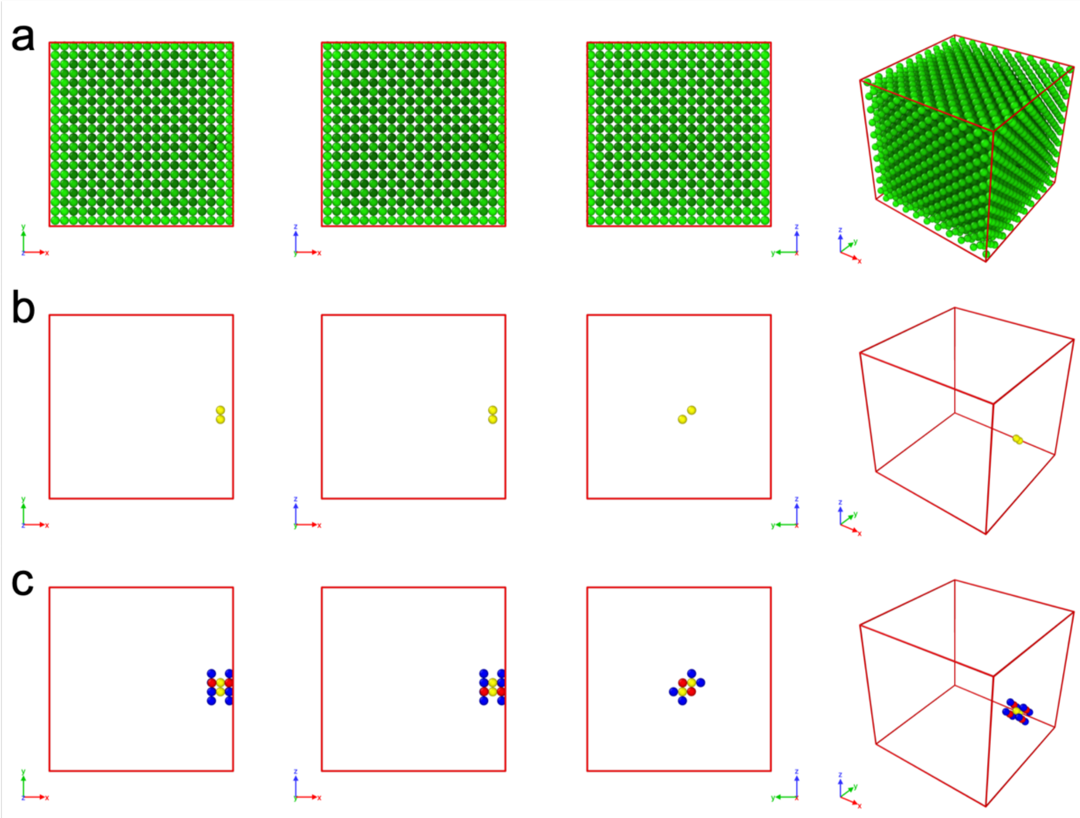

Supplementary Figure 9 | Sensitivity analysis result for unit cell with 3, 988 real atoms and 2 virtual atoms. a, Visualizations of real atoms (green) from different perspectives. **b,** Visualizations of virtual atoms (yellow) from different perspectives. **c,** Visualizations of the 14 atoms with the lowest sensitivity values from different perspectives. The two atoms (yellow) with the lowest sensitivity value are the virtual atoms. Due to the symmetry of the crystal structure, there are four atoms (red) with the second-lowest sensitivity value and eight atoms (blue) with the third-lowest sensitivity value.

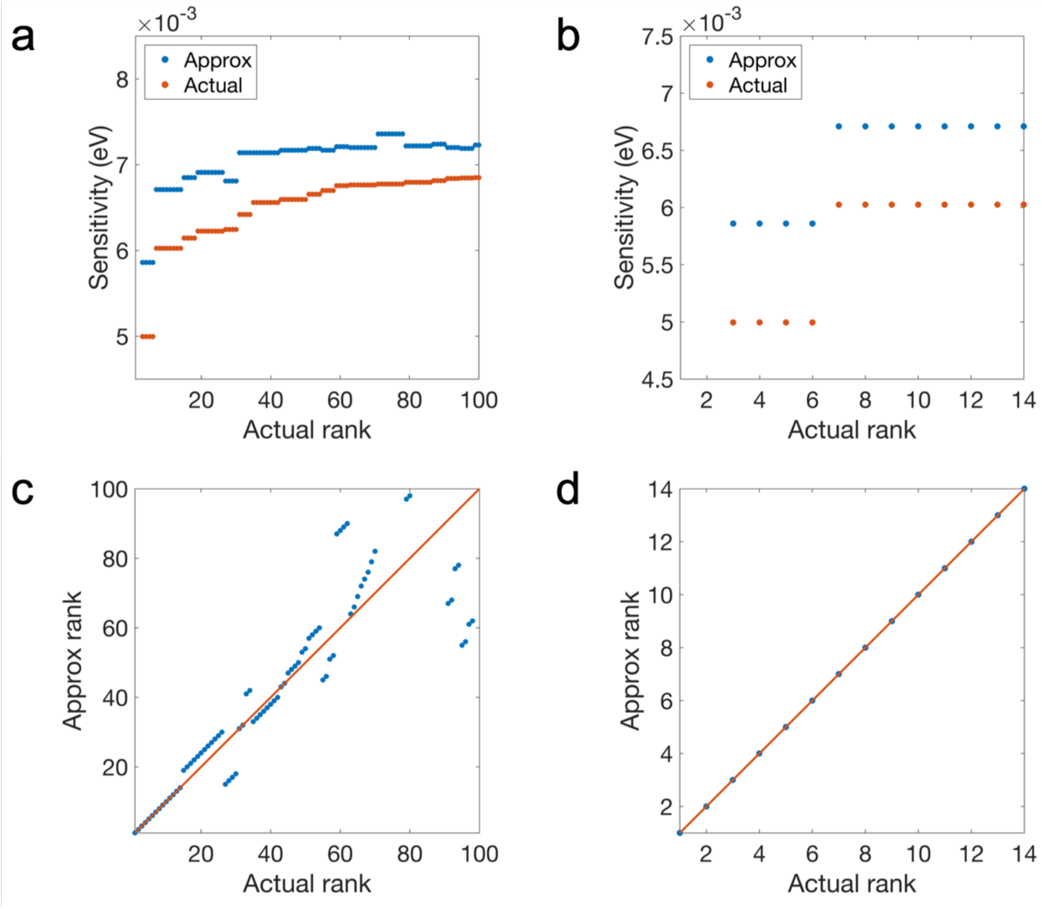

Supplementary Figure 10 | Comparison of sensitivity analysis results. a, A comparison of the sensitivity values calculated using the sensitivity approximation (blue dots) and those calculated using the one-at-a-time technique (red dots) for the 100 atoms with the lowest sensitivity values. **b,** A comparison of the sensitivity values for the 14 atoms with the lowest sensitivity values. Note that the rank-1 and rank-2 atoms are the virtual atoms with a sensitivity value of zero. **c,** A comparison of the sensitivity ranks calculated using the sensitivity approximation and those calculated using the one-at-a-time technique for the 100 atoms with the lowest sensitivity values. **d,** A comparison of the sensitivity ranks for the 14 atoms with the lowest sensitivity values.

Supplementary Tables

Supplementary Table 1 | Relative densities of TPMS structures based on solid-networks versus surface threshold.

Gyroid			Diamond			Primitive		
t	atoms	density	t	atoms	density	t	atoms	density
0.0	2,003	0.5008	0.0	2,016	0.5040	0.0	2,000	0.5000
-0.1	2,100	0.5250	-0.1	2,208	0.5520	-0.1	2,072	0.5180
-0.2	2,268	0.5670	-0.2	2,400	0.6000	-0.2	2,180	0.5450
-0.3	2,400	0.6000	-0.3	2,592	0.6480	-0.3	2,276	0.5690
-0.4	2,520	0.6300	-0.5	2,784	0.6960	-0.4	2,516	0.6290
-0.5	2,640	0.6600	-0.6	3,168	0.7920	-0.6	2,636	0.6590
-0.6	2,760	0.6900				-0.8	2,816	0.7040
-0.7	2,916	0.7290				-0.9	3,092	0.7730
-0.8	3,036	0.7590				-1.0	3,192	0.7980
-0.9	3,212	0.8030						

Supplementary Table 2 | Lattice properties of aluminum in present work and reference[1].

	Present work	Reference
a_0 (nm)	0.405	0.405
E_0(eV/atom)	-3.36	-3.36
K(GPa)	79	79
C_{11}(GPa)	114	114
C_{12}(GPa)	61.5	61.6
C_{44}(GPa)	31.6	31.6

Supplementary Methods

Elastic properties calculations: Periodic boundary conditions are imposed by using the style p boundary command in LAMMPS (http://lammps.sandia.gov). Consequently, each atom in the unit cell not only interacts with the other atoms in the same unit cell but also with their mirror images in the adjacent unit cells. To calculate the elastic properties of a material system (e.g., Nano-TO design, gyroid structure), the system is first relaxed to reach the equilibrium state (stress-free). An energy minimization using the conjugate gradient (CG) algorithm is performed to equilibrate the system. During the energy minimization, the simulation box is allowed to adjust the size and the atoms are allowed to move to reduce the total energy of the system. After the equilibrium state is reached, a small negative strain (-10^{-3}) is applied along the x-direction ($\varepsilon_{xx} = -10^{-3}$) and all other strains are set to be zero. In this deformed state, the corresponding stress tensor is computed after the system is relaxed by another energy minimization. As the strains of the system are fixed, the simulation box is not allowed to adjust the size, however, the atoms are still allowed to move to reduce the total energy of the system during the energy minimization. The elastic constant of C_{11} can be calculated from the stress tensor as the ratio between the stress and the applied strain along the x-direction. The elastic constant of C_{11} can also be calculated from another stress tensor by applying a small positive strain (10^{-3}) along the x-direction ($\varepsilon_{xx} = 10^{-3}$). The elastic constants are formally defined in terms of the second derivative of the free energy with respect to strain evaluated at zero strain. By definition, these are independent of the sign of the strain. However, in our computational approach, we apply finite strains to evaluate the elastic constants. The implication is that higher order terms in the Taylor series defining the free energy at this strain also contribute to the observed stress. This can lead to small differences depending on the sign of the imposed strain. For instance, when a strain of 10^{-3} is applied to calculate the elastic constants of aluminum, the elastic constant of C_{11} is calculated as 113.94 GPa in compression and 113.57 GPa in tension. The values are very close, and the difference is only 0.3%. To average these values, we fit a line to the stress–strain curve, and the slope of this line is

used to determine the elastic constant of interest. Furthermore, we find that the elastic constants thus determined are insensitive to the magnitude of the applied strain when the strain is small enough (smaller than 10^{-2}). For instance, the elastic constant of C_{11} (mean value) is calculated as 113.76 GPa for aluminum when the applied strain is 10^{-3}. This value becomes 113.79 GPa when the applied strain is decreased to 10^{-4} or 113.39 GPa when the applied strain is increased to 10^{-2}. The other elastic constants (C_{ij}) are calculated using the same approach.

Random initialization: To ensure that the optimized designs are of high performance, Nano-TO is performed multiple times starting with different initial structures. The only difference between those initial structures is the location of the initially assigned virtual atom. Although those initial structures are physically identical due to the symmetry of the crystal structure and periodic boundary conditions, they would lead to different optimized designs. The reason is that multiple atoms would have the same sensitivity value during the optimization process. The ranking of those atoms with the same sensitivity value will retain their initial order (atom index) in the sensitivity analysis. Consequently, different initial structures would lead to different optimization paths and converge to different optimized designs.

Supplementary Notes

Objective of maximizing the elastic constant of C_{33}: Here we apply Nano-TO to design nanostructured materials with the objective of maximizing the elastic constant of C_{33}. The elastic constant of C_{33} represents the resistance of a material to being elastically deformed in the *z*-direction (vertical direction). The purpose of this case study is to validate the reliability of Nano-TO as the optimal structures for maximizing the elastic constant of C_{33} with a volume fraction constraint are relatively intuitive. The design domain is a cubic unit cell with a length of approximately 4 nm. Periodic boundary conditions are imposed along the *x*-, *y*-, and *z*-directions to create the supercell structure for evaluating the macroscopic elastic properties. The optimization parameters used here are the same as those used in the case study with the objective of maximizing the bulk modulus in the main paper. Starting with different initial structures, a total of 16 designs with a volume fraction of 50% are generated by Nano-TO. Those designs have the elastic constant of C_{33} in a range of 38.54 to 43.51 GPa, with an average of 41.95 GPa. The design with the highest elastic constant of C_{33} is denoted by Nano-TO design and selected for further examination. The Nano-TO designs with varying volume fractions are shown in Supplementary Figs. 2a and 2b. Periodic images of the Nano-TO design with a volume fraction of 50% are shown in Supplementary Figs. 2c and 3. The structural evolution during the optimization process is presented in Supplementary Movies 3 and 4.

The optimal structures for maximizing the elastic constant of C_{33} are expected to be similar to those for maximizing the Young's modulus in the *z*-direction. It can be seen in the figures that the virtual atoms form pillar-like structures in parallel to the *z*-direction. The surfaces in the Nano-TO designs are mostly $\{1\ 0\ 0\}$. As expected, the Nano-TO designs are the optimal designs for maximizing the Young's modulus, in which the real and virtual materials are connected side-by-side, in parallel to the deformation direction. For comparison, the Young's modulus of the Nano-TO design with a volume fraction of 50% is

34.20 GPa in the z-direction. However, based on the rule of mixtures (Voigt model), the maximum Young's modulus for the same volume fraction should be 35.29 GPa, which is 50% of the Young's modulus of the base material (70.58 GPa). This theoretical upper bound is slightly higher than the Young's modulus of the Nano-TO design due to the surface effect at the nanoscale.

To study the surface effect on the elastic properties of the Nano-TO design, an atomistic model of a nanoplate with a free surface $\{1\ 0\ 0\}$ on each side is created and shown in Supplementary Fig. 4a (left). Periodic boundary conditions are imposed along the in-plane directions (i.e., $[0\ 1\ 0]$ and $[0\ 0\ 1]$). Vacuum regions are created for both sides of the nanoplate in the $[1\ 0\ 0]$ direction to represent two flat free surfaces. In this specific model, the nanoplate consists of 12 layers including 2 surface layers and 10 bulk layers. Other models with varying numbers of layers from 4 to 96 are also created. The thickness of those models is in a range of 0.81 to 19.44 nm. For comparison, another atomistic model of a nanoplate with a free surface $\{1\ 1\ 0\}$ on each side is created and shown in Supplementary Fig. 4a (middle). Similarly, periodic boundary conditions are imposed along the in-plane directions (i.e., $[\bar{1}\ 1\ 0]$ and $[0\ 0\ 1]$). Vacuum regions are created for both sides of the nanoplate in the $[1\ 1\ 0]$ direction to represent two flat free surfaces. In this specific model, the nanoplate consists of 8 layers including 2 surface layers and 6 bulk layers. Other models with varying numbers of layers from 4 to 64 are also created. The thickness of those models is in a range of 1.15 to 18.33 nm. In addition to those two low-index surfaces, $\{1\ 0\ 0\}$ and $\{1\ 1\ 0\}$, there are oblique surfaces which are also perpendicular to the z-direction (i.e., $[0\ 0\ 1]$). Those oblique surfaces are more complex and often stepped surfaces. To consider oblique surfaces, an atomistic model of a nanoplate with a free surface $\{2\ 1\ 0\}$ on each side is created and shown in Supplementary Fig. 4a (right). Similarly, periodic boundary conditions are imposed along the in-plane directions (i.e., $[\bar{1}\ 2\ 0]$ and $[0\ 0\ 1]$). Vacuum regions are created for both sides of the nanoplate in the $[2\ 1\ 0]$ direction to represent two free surfaces. In this specific model, the thickness is 2.72 nm. Other models with varying

thicknesses are also created. The thickness of those models is in a range of 0.91 to 18.11 nm.

The modulus ratios in the z-direction for the nanoplates with varying thicknesses are shown in Supplementary Fig. 4b. The modulus ratio is defined as the Young's modulus of a nanoplate divided by that of the bulk material. The surface $\{1\ 0\ 0\}$ is stiffer than the surfaces $\{1\ 1\ 0\}$ and $\{2\ 1\ 0\}$ when the thickness is small. This result might explain why the surfaces in the Nano-TO designs are mostly $\{1\ 0\ 0\}$. As we only consider one type of oblique surface (i.e., $\{2\ 1\ 0\}$), future studies are required to confirm whether the surface $\{1\ 0\ 0\}$ is indeed the stiffest surface among all the possible surfaces (e.g., $\{3\ 1\ 0\}$, $\{3\ 2\ 0\}$) perpendicular to the z-direction. It can be seen in the figure that the surfaces $\{1\ 0\ 0\}$, $\{1\ 1\ 0\}$, and $\{2\ 1\ 0\}$ are all softer than the bulk material due to the lower atomic coordination. Note that whether a surface is stiffer or softer than the bulk material depends on the competition between the electron redistribution and the lower coordination on surfaces[2]. Here we take the Nano-TO design with a volume fraction of 50% as a template and parametrize the design to create the same design with varying cell sizes. The Nano-TO designs with varying cell sizes from 4 to 64 nm are created and their Young's moduli are shown in Supplementary Fig. 4c. The Young's modulus of the Nano-TO design increases with the cell size and converges to the theoretical upper bound (35.29 GPa).

TPMS structures based on sheet-networks: TPMS structures can be created by two main strategies based on either solid-networks or sheet-networks[3]. The first strategy (solid-networks) is by in-filling one side of the surface and the volume fraction is controlled by the threshold of the surface. The second strategy (sheet-networks) is by giving the surface a certain thickness and the volume fraction is controlled by the thickness of the surface. For a given volume fraction, TPMS structures based on sheet-networks have a larger surface-to-volume ratio than those based on solid-networks. For small unit cells (e.g., 4 nm), TPMS structures based on sheet-networks are less stable due to a larger surface-to-volume ratio. For this reason, we compare the Nano-TO designs with the TPMS structures based on solid-networks in the main

paper. To be able to compare the Nano-TO designs with TPMS structures based on sheet-networks, here we only consider larger unit cells (at least 16 nm). It has been shown that TPMS structures based on sheet-networks are mechanically superior than those based on solid-networks especially at relatively low volume fractions[3]. Therefore, instead of considering the volume fraction of 50% as in the main paper, we reduce the volume fraction to 25%. The Nano-TO design with a volume fraction of 25% is created by adjusting the size of the vacancies with truncated octahedron structures in the BCC arrangement. TPMS structures including the gyroid (G), diamond (D), and primitive (P) based on sheet-networks are considered. The Nano-TO design and TPMS structures with different cell sizes from 16 to 64 nm are created (Supplementary Fig. 7) and their bulk moduli are shown in Supplementary Figs. 8a and 8b. The result shows that the bulk modulus of the Nano-TO design is always higher than those of the TPMS structures (sheet-networks) regardless of the cell size. The bulk modulus of the Nano-TO design exceeds the HS upper bound when the cell size is small and converges to the HS upper bound when the cell size is large enough. For instance, when the cell size is 16 nm, the bulk modulus of the Nano-TO design with a volume fraction of 25% is 8.56 GPa and the HS upper bound for the same volume fraction is only 7.84 GPa.

Furthermore, the atomic strain (ε_{zz}) distributions of those structures subjected to a constant hydrostatic (volumetric) strain of -10^{-2} are shown in Supplementary Fig. 8c. The cell size in the comparison is 16 nm and the volume fraction is 25%. The average atomic strains for the Nano-TO design, gyroid, diamond, and primitive are -0.0039, -0.0031, -0.0029, and -0.0031, respectively. The average atomic strains of ε_{xx} and ε_{yy} are the same as that of ε_{zz} due to the symmetry of those structures. The result shows that the Nano-TO design has a better load transfer mechanism as the average atomic strain (compressive) is larger than those of the TPMS structures. Consequently, a higher bulk modulus is achieved as more elastic strain energy can be accommodated.

Sensitivity approximation: To perform a sensitivity analysis with a reasonable computational cost, the gradient of the objective function (elastic strain energy) with respect to the design variable x_k is approximated as two times that of its elastic strain energy (see Methods). Compared to using a one-at-a-time technique that evaluates the sensitivity value for one atom at a time while keeping the other atoms fixed, this sensitivity approximation allows the evaluation of sensitivity values for all atoms at the same time. To estimate the accuracy of this sensitivity approximation, a cubic unit cell with $4{,}000$ atoms is created. Among the $4{,}000$ atoms, $3{,}988$ of them are set to be real atoms and the other 2 are set to be virtual atoms in the initial structure. The real and virtual atoms in the unit cell are shown in Supplementary Figs. 9a and 9b, respectively. Here we apply Nano-TO to maximize the bulk modulus starting with this initial structure. In this case, maximizing the bulk modulus is equivalent to maximizing the elastic strain energy of the material system subjected to a constant hydrostatic (volumetric) strain. A sensitivity analysis using the sensitivity approximation is performed to identify the real atoms with the lowest sensitivity values (to be converted to virtual atoms). The 14 atoms with the lowest sensitivity values are shown in Supplementary Fig. 9c. Among the 14 atoms, 2 of them have the lowest sensitivity value (0 eV). These two atoms (yellow) are considered the most inefficient. However, these two atoms are the two virtual atoms in the initial structure (trivial result). Thus, the atoms with the second-lowest sensitivity value (0.0059 eV) are identified. Due to the symmetry of the crystal structure, there are four atoms (red) with this sensitivity value. The atoms with the third-lowest sensitivity value (0.0067 eV) are also identified. There are eight atoms (blue) with this sensitivity value.

To evaluate the accuracy of the sensitivity analysis result using the sensitivity approximation (Supplementary Fig. 9c), another sensitivity analysis is performed using a one-at-a-time technique. Specifically, the sensitivity value of an atom is calculated as the difference between the elastic strain energy of the material system with the atom and that without the atom. The sensitivity analysis result obtained using the one-at-a-time technique is considered exact since there is no approximation applied in

the calculation. Comparisons of the sensitivity values calculated using the sensitivity approximation and those calculated using the one-at-a-time technique are shown in Supplementary Figs. 10a and 10b. Although the absolute sensitivity values do not match well, the relative values are comparable. Note that the absolute sensitivity values are not important for Nano-TO. The relative values are more important as the ranking of sensitivity is used to determine which real atoms need to be converted to virtual atoms and vice versa (see Methods). Comparisons of the sensitivity ranks calculated using the sensitivity approximation and those calculated using the one-at-a-time technique are shown in Supplementary Figs. 10c and 10d. It can be seen in the figures that the sensitivity ranks match very well, especially for those atoms with the highest ranks (lowest sensitivity values). The 14 atoms with the lowest sensitivity values identified using the sensitivity approximation and those identified using the one-at-a-time technique are identical (Supplementary Fig. 10d). Therefore, we believe that the sensitivity approximation adopted in this study is accurate enough for identifying atoms with the lowest sensitivity values.

Supplementary References

1. Mishin, Y., Farkas, D., Mehl, M. & Papaconstantopoulos, D. Interatomic potentials for monoatomic metals from experimental data and ab initio calculations. *Physical Review B* **59**, 3393 (1999).
2. Zhou, L. & Huang, H. Are surfaces elastically softer or stiffer? *Applied Physics Letters* **84**, 1940-1942 (2004).
3. Al-Ketan, O., Al-Rub, R. K. A. & Rowshan, R. Mechanical properties of a new type of architected interpenetrating phase composite materials. *Advanced Materials Technologies* **2**, 1600235 (2017).
