# Supplementary material for: Nano-topology optimization for materials design with atom-by-atom control
Source: Nat Commun. 2020 Jul 27;11:3745. doi: 10.1038/s41467-020-17570-1 (PMC7385150; doi:10.1038/s41467-020-17570-1)
Supplement: Supplementary file 3 — Description of Additional Supplementary Files [file 41467_2020_17570_MOESM3_ESM.docx]

File Name: Supplementary Movie 1

Description: The structural evolution of real atoms during the optimization process for maximizing the bulk modulus.

File Name: Supplementary Movie 2

Description: The structural evolution of virtual atoms during the optimization process for maximizing the bulk modulus.

File Name: Supplementary Movie 3

Description: The structural evolution of real atoms during the optimization process for maximizing the elastic constant of *C_33_*.

File Name: Supplementary Movie 4

Description: The structural evolution of virtual atoms during the optimization process for maximizing the elastic constant of *C_33_*.
